# Climate-smart agricultural practices influence the fungal communities and soil properties under major agri-food systems

**DOI:** 10.3389/fmicb.2022.986519

**Published:** 2022-12-13

**Authors:** Madhu Choudhary, Hanuman S. Jat, Mangi L. Jat, Parbodh C. Sharma

**Affiliations:** ^1^Indian Council of Agricultural Research-Central Soil Salinity Research Institute (ICAR-CSSRI), Karnal, India; ^2^International Maize and Wheat Improvement Center (CIMMYT), New Delhi, India; ^3^International Crops Research Institute for the Semi-Arid Tropics (ICRISAT), Patancheru, India

**Keywords:** agriculture management, tillage, fungal community, diversity indices, climate smart agricultural practices, soil organic carbon

## Abstract

Fungal communities in agricultural soils are assumed to be affected by climate, weather, and anthropogenic activities, and magnitude of their effect depends on the agricultural activities. Therefore, a study was conducted to investigate the impact of the portfolio of management practices on fungal communities and soil physical–chemical properties. The study comprised different climate-smart agriculture (CSA)-based management scenarios (Sc) established on the principles of conservation agriculture (CA), namely, ScI is conventional tillage-based rice–wheat rotation, ScII is partial CA-based rice–wheat–mungbean, ScIII is partial CSA-based rice–wheat–mungbean, ScIV is partial CSA-based maize–wheat–mungbean, and ScV and ScVI are CSA-based scenarios and similar to ScIII and ScIV, respectively, except for fertigation method. All the scenarios were flood irrigated except the ScV and ScVI where water and nitrogen were given through subsurface drip irrigation. Soils of these scenarios were collected from 0 to 15 cm depth and analyzed by Illumina paired-end sequencing of Internal Transcribed Spacer regions (ITS1 and ITS2) for the study of fungal community composition. Analysis of 5 million processed sequences showed a higher Shannon diversity index of 1.47 times and a Simpson index of 1.12 times in maize-based CSA scenarios (ScIV and ScVI) compared with rice-based CSA scenarios (ScIII and ScV). Seven phyla were present in all the scenarios, where Ascomycota was the most abundant phyla and it was followed by Basidiomycota and Zygomycota. Ascomycota was found more abundant in rice-based CSA scenarios as compared to maize-based CSA scenarios. Soil organic carbon and nitrogen were found to be 1.62 and 1.25 times higher in CSA scenarios compared with other scenarios. Bulk density was found highest in farmers' practice (Sc1); however, mean weight diameter and water-stable aggregates were found lowest in ScI. Soil physical, chemical, and biological properties were found better under CSA-based practices, which also increased the wheat grain yield by 12.5% and system yield by 18.8%. These results indicate that bundling/layering of smart agricultural practices over farmers' practices has tremendous effects on soil properties, and hence play an important role in sustaining soil quality/health.

## Introduction

Rice–wheat (RW) system is the dominant cropping system of the Indo-Gangetic Plains of South Asia, spreading over a 13.5 M ha area (Gupta and Seth, 2007). In this system, farmers follow conventional agricultural practices, such as repeated tillage, open field residue burning, indiscreet use of fertilizers, and irrigation waters, which lead to soil health deterioration, groundwater depletion, and environmental pollution (Jat et al., 2021a). In the 21st century, climate change with extreme weather events is likely to be the most serious issue being faced by mankind as per the IPCC predictions (IPCC, 2022). To adapt to extreme events and to manage natural resources, climate-smart agriculture (CSA)-based management practices on the principles of conservation agriculture (CA) (zero tillage, residue retention, and crop rotation) and mitigation co-benefits may prove an excellent option for conventional agriculture to maintain the sustainability of soil and cropping system. In CSA practices, the conventional management practices switch over to resource-conserving management practices by layering with zero/no-tillage, residue retention/incorporation, crop diversification, precise irrigation water, and nutrient management practices to sustain soil and farm productivity and environmental quality (Choudhary et al., 2020). In CSA, residues are managed through retention or incorporation, which are burnt by the farmers under conventional management practices. Crop residues provide an ambient environment for soil fungi, but their density and diversity depend upon the type of residue served. Different types of crop rotations provide different types of root exudates, which influence microbial community compositions (Huang et al., 2014; Jiang et al., 2016). Crop rotation and tillage practices disturb soils and influence the distribution of microbes in the soil layer (Orrù et al., 2021). Fungi play a great role in agricultural practices in nutrient cycling, transformation, and availability.

Soil is the base of all civilizations and mankind, therefore, its functions and structure, directly and indirectly, influence food security. Soil functions are governed by climatic, edaphic, and anthropogenic activities. Soil properties and composition of the soil microbiota are directly interrelated with each other (Wakelin et al., 2008; Bender et al., 2016). Soil microbial communities are essential to biogeochemical cycles, and especially fungi play an important role in the biodegradation of organic matter (Rineau et al., 2012; Qiu et al., 2018). They dominate the microbial biomass in soil habitats (Joergensen and Wichern, 2008) and contribute to nutrient cycling (Stromberger, 2005). Soils have dynamic environments and the microorganisms that live in these soil habitats respond to the changing soil conditions. It has been found that more genera and species of fungi exist in the soil than in any other environment (Nagmani et al., 2006). Fungi are the primary decomposers in soils and secrete various enzymes, such as cellulases, laccases, and xylanases that break down lignocelluloses into simple sugars (Maza et al., 2014; Choudhary et al., 2016). A comprehensive understanding of the fungal community in agricultural soils provides the path to study their roles in the soil ecosystem. The relationship between the biodiversity of soil fungi and ecosystem function is an important issue, particularly in the concern of global climate change and human alteration of ecosystem processes. Fungi play an important role in soil formation, structure, and fertility by contributing to the nutrient cycle and maintenance of the ecosystem (Stromberger, 2005; Hoorman, 2011). Agricultural practices that impact soil conditions can significantly alter soil microbial community composition (Choudhary et al., 2018a,b). The differences in microbial community composition are mainly been attributed to differences in the soil properties (physical and chemical), which are linked to agricultural management practices (IPCC, 2022). Some studies reported the effects of different tillage practices on soil chemical and physical properties and microbial activities, and they reported better soil properties under zero tillage (ZT) and residue retention practices (Choudhary et al., 2018c,d). Agricultural management practices have an impact on different parameters of soil. Under CA practices, better soil physical properties such as lower bulk density (Li et al., 2019), enhanced saturated hydraulic conductivity (Patra et al., 2019), higher infiltration rate (Jat et al., 2018), and increased water-stable aggregates (Bhattacharyya et al., 2012; Jat et al., 2019a) were reported than conventional management practices in cereal-based systems. Increased nutrient availability (Zahid et al., 2020), soil organic carbon (Bera et al., 2017), and nitrogen (Bhattacharyya et al., 2018) were favored under no-tillage/ZT and residue management-based practices. In the western Indo-Gangetic Plains, higher soil biological properties and soil health were reported with CA-based management practices (Choudhary et al., 2018c,d; Sharma et al., 2021).

In any agriculture system, different soil processes depend on microbial community compositions. The diverse nature of fungal communities makes them an important factor for sustainable agriculture in the changing scenarios of climate change (Yadav et al., 2020). The impact of different agricultural management regimes on fungal community composition gained rising interest, although up to date, only a few studies were dedicated to determining the effects of tillage, fertilization, and crop rotation on microbial diversity in cereal systems (Sharma-Poudyal et al., 2017; Choudhary et al., 2018b; Piazza et al., 2019). To date, some studies have applied next-generation sequencing techniques in South Asia to investigate the influence of different tillage regimes and types of crop residue management on soil fungal communities (Choudhary et al., 2018a,b). Our study focused on the changes in the fungal communities under the layering of different management practices, such as soil tillage (conventional vs. zero tillage), residue management (without residue vs. residue retention), crop rotation (rice–wheat vs. maize–wheat), and irrigation (border vs. subsurface drip). The ultimate objective was to identify the impact of layering of management practices on the composition of soil fungal communities.

## Materials and methods

### Study location, experimental design, and management

**Figure 1:**
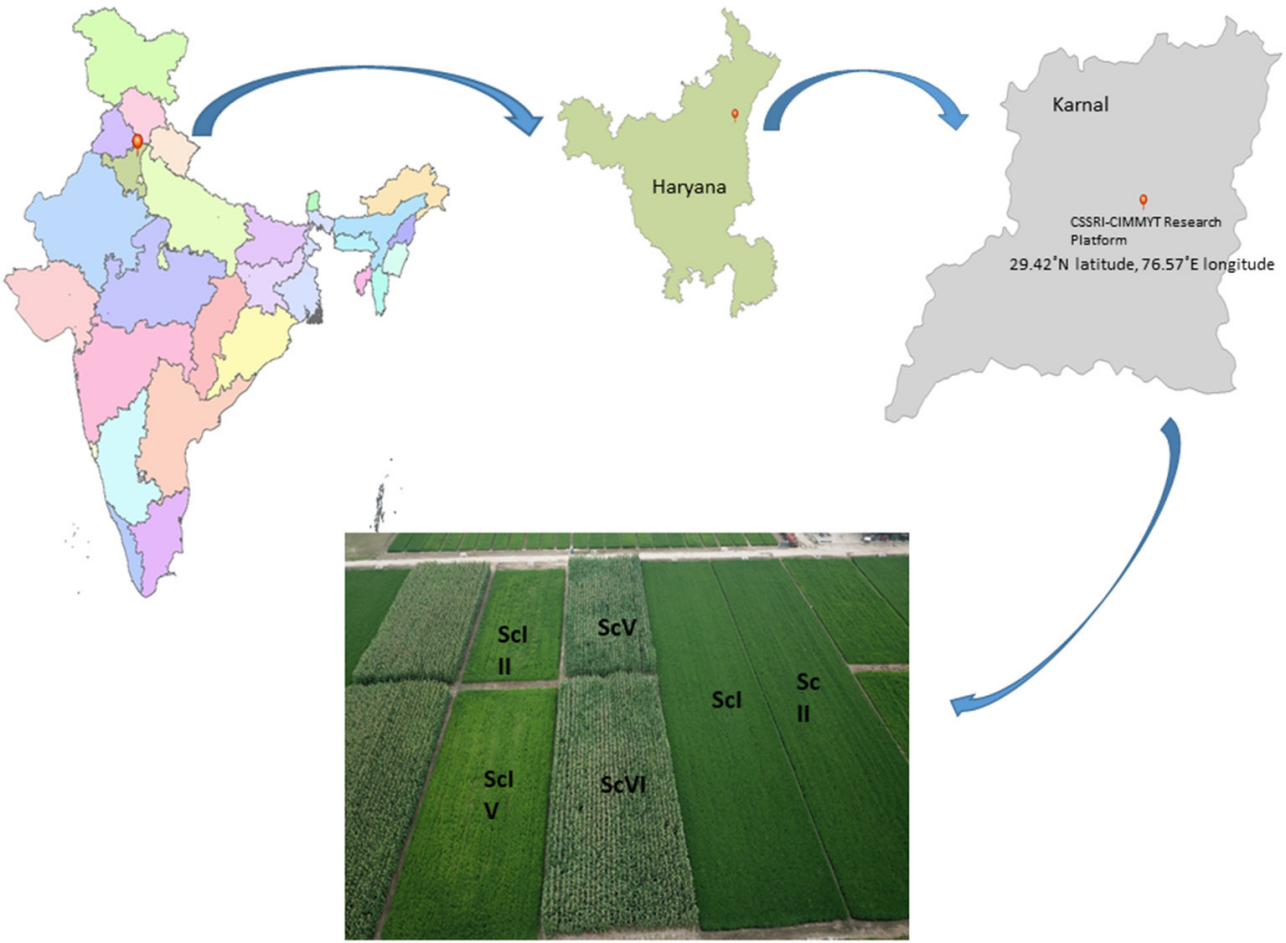
Different cereal-based management scenarios at CSSRI-CIMMYT research platform, Karnal, Harayana, India.

A 9-year (2009–2018) long-term production scale fixed plot experiment was conducted at the Indian Council of Agricultural Research, Central Soil Salinity Research Institute (29.42°N latitude, 76.57°E longitude, and at an elevation of 243 m.a.s.l.), Karnal, India. The soil of the experimental field is silty loam in texture and falls under the *Typic Natrustalf* category (Soil Survey Division Staff, 1993). The experiment included six cereal-based scenarios varied with residue, tillage, crop, irrigation, and nutrient supply (Figure 1). The treatments are described as scenarios because they are varied in multiple indicators, namely, tillage, crop establishment, residue management, irrigation management, system intensification, etc. Initially, the experiment started with four scenarios, and later on, in 2016, two more scenarios (ScV and ScVI) were added (Choudhary et al., 2020; Jat et al., 2021b) with minor changes in ScIII and ScIV, and only irrigation management was precise (subsurface drip irrigation was used instead of border irrigation). The details of all the scenarios along with their management practices are presented in Table 1. Scenario III and ScIV were based on the principles of CA practices, so they were described as partial CSA. However, in ScV and ScVI, in addition to ScIII and ScIV, irrigation water and N were precisely managed using subsurface drip irrigation (SDI) and called full CSA. Scenarios were structured in a randomized complete block design and replicated three times. In ScI (farmers' practice or business as usual), both rice and wheat were established with conventional practice. Rice was planted by manual transplanting with 25–30 days old seedlings in puddled fields and wheat was planted by manual broadcasting in tilled soil under ScI. In ScII, manual transplanting for rice in a random geometry (20 × 15 cm) was done. In other scenarios (zero tillage (ZT) conditions), all the crops (rice, wheat, and mungbean) were planted with a row spacing of 22.5 cm using Happy Seeder with an inclined plate seed metering mechanism. However, maize was seeded by Happy Seeder at a row spacing of 67.5 cm. In the farmers' practice (ScI), all the crop residues of rice and wheat were removed from the ground level. However, in other scenarios, it was managed as per the details presented in Table 1. The total amount of crop residue in different scenarios ranged from 100.5 to 119.25 Mg ha^−1^ in 9 years of study (Table 1). Maize-based scenario received the highest (~119 Mg ha^−1^) amount of crop residue, while the rice was based on the range of 100–105 Mg ha^−1^. The NPK dose (nitrogen, phosphorus, and potash) was given as per the recommendation of CCS Haryana Agricultural University for all the crops. In the subsurface drip-irrigated scenario (ScV and ScVI), 80% of the total N as urea (minus N added through DAP and NPK complex) was applied through irrigation (fertigation). In rice, maize, and wheat, irrigation was applied based on soil moisture potential (SMP) using a tensiometer.

**Table 1:** Description of different cereal-based management scenarios.

**Scenarios**	**Crop rotations**	**Tillage**	**Crop establishment method**	**Residue management options**	**Water management options**	**Residue load**
ScI	Rice–Wheat–Fallow	CT–CT	Rice: Transplanting Wheat: Broadcast	All residue removed	Border irrigation	–
ScII	Rice–Wheat–Mungbean	CT–ZT–ZT	Rice: Transplanting Wheat: Drill seeding Mungbean: Drill/relay	Full (100%) rice and anchored wheat residue retained on soil surface; full mungbean residue incorporated	Border irrigation	104.67^b^
ScIII	Rice–Wheat–Mungbean	ZT–ZT–ZT	Rice: Drill seeding Wheat: Drill seeding Mungbean: Drill/relay	Full (100%) rice and mungbean; anchored wheat residue retained on soil surface	Border irrigation	100.5^c^
ScIV	Maize–wheat–Mungbean	ZT–ZT–ZT	Maize: Drill seeding Wheat: Drill seeding Mungbean: Drill/relay	Maize (65%) and full mungbean; anchored wheat residue retained on soil surface	Border irrigation	119.25^a^
ScV	Rice–Wheat–Mungbean	ZT–ZT–ZT	Same as in scenario 3	Same as in scenario 3	Subsurface drip irrigation	100.95^c^
ScVI	Maize–wheat–Mungbean	ZT–ZT–ZT	Same as in scenario 4	Same as in scenario 4	Subsurface drip irrigation	119.11^a^

a, b, c Means followed by similar lowercase letters within a column are not significantly different at 0.05 level of probability using Tukey's HSD test.

### Soil sampling

The soil samples were collected from all six scenarios (three from each scenario) at 0–15 cm soil depth after harvesting of wheat crop in May 2018. The soil surface was cleaned by removing crop residues and samples were taken randomly aseptically using an auger. Collected soil samples (18 samples) were sieved with 2 mm mesh to eliminate large soil aggregates and plant roots. Samples were divided into three parts, one part was immediately transferred to the laboratory, stored at −20°C until DNA was extracted, and the second part is stored in the refrigerator at 4°C for further analysis of biological properties. The third part was air-dried, ground, and stored in glass containers for chemical and physical analysis.

### DNA extraction, amplification, and sequencing

From the soil samples, DNA was extracted by MO BIO's PowerSoil^®^ DNA Isolation Kit as per the instructions of the manufacturer. The quantity and quality of DNA were measured by Nanodrop spectrophotometer (Thermo Fisher Scientific, USA) and agarose gel electrophoresis, respectively. ITS1 and ITS2 sequencing libraries were constructed by a two-step PCR-based workflow. For round one PCR, using a template of 10–100 ng metagenomic DNA, ITS1 and ITS2 regions were amplified using region-specific proprietary primers developed at Genotypic Technology Pvt. Ltd., Bangalore, India. The protocol also includes overhang adapter sequences that were appended to the primer pair sequences for compatibility with Illumina index and sequencing adapters. PCR was carried out for 26 cycles (hot-start 95°C/3 min, denaturation 95°C/30 s, annealing 55°C/30 s, extension 68°C/1 min, elongation 68°C/5 min, and infinite hold at 4°C) using 0.5 μM primers. The resultant amplicons were analyzed on 1.5% agarose gel whereby the desired amplicons were observed to be of sizes ~350–450 bp for ITS1 and ~450–500 bp for ITS2. For the second round of PCR, 1 μl of 1:2 diluted round one PCR amplicons were taken and amplified for 10 cycles to add Illumina sequencing barcoded adaptors (Nextera XT v2 Index Kit, Illumina, USA). Second round PCR amplicons (sequencing libraries) were analyzed on 1.5% agarose gel, then purified using Ampure XP magnetic beads (Beckman Coulter, USA), and concentrations were measured using Qubit dsDNA HS assay (Thermo Fisher Scientific, USA). The ITS1 and ITS2 amplicons were generated separately by individual PCRs and libraries were constructed off these amplicons individually. The sample libraries were normalized based on the qubit concentrations, then multiplexed on the MiSeq flowcell, and sequenced using a 300PE read length chemistry.

The forward and reverse primers (White et al., 1990; Fujita et al., 2001) were as follows:

ITS1_Forward TCCGTAGGTGAACCTGCGG.
ITS1_Reverse GCTGCGTTCTTCATCGATGC.
ITS2_Forward GCATCGATGAAGAACGCAGC.
ITS2_Reverse TCCTCCGCTTATTGATATGC.

We have adapted our primer design based on fungal rDNA sequencing literature targeting the conserved regions of 5.8S rDNA and 28S rDNA. ITS1 primers were used to amplify the intervening 5.8S rDNA and the adjacent ITS1 region. ITS2 primers were used to amplify a longer region across the 5.8S rDNA through the intervening ITS2 region and the large 28S subunit.

### Analysis of soil properties

The organic carbon (OC) content of the soils was determined using the wet oxidation method (Walkley and Black, 1934). The available nitrogen (N) in soil was determined by the alkaline permanganate method of Subbiah and Asija (1956), available phosphorus (P) by the ascorbic acid reductant method of Olsen et al. (1954), and available potassium (K) by flame photometer using neutral 1N ammonium acetate extractant as described by Jackson (1973). Mean weight diameter (MWD) and water-stable aggregates (WSA) were determined following the method of Jat et al. (2019b). Soil bulk density (BD) was measured by core sampler method (Blake and Hartge, 1986).

### Crop yield

At maturity, crops were harvested manually for grain and straw yields according to residue management protocols. Grain yield was expressed as Mg ha^−1^ at 14, 12, and 14% grain moisture content for rice, wheat, and maize, respectively. Grain yields of maize, rice, wheat, and mungbean were converted to the rice equivalent yield (REY) by using Equation 1.


(1) $$\begin{array}{l} \text{Rice equivalent yield }\left(\text{Mg }{\text{ha}}^{\text{−1}}\right)\text{=}[\text{maize/wheat/mungbean yield}\\ \;\left(\text{Mg }{\text{ha}}^{\text{−1}}\right)\;\times \text{MSP of respective crop }\left(\text{INR }{\text{Mg}}^{\text{−1}}\right)]/\\ \;\left[\text{MSP of rice }\left(\text{INR }{\text{Mg}}^{\text{−1}}\right)\right] \end{array}$$

where, MSP, Minimum support price of Govt. of India; INR, Indian Rupee.

### Bioinformatics and statistical analysis

The Illumina paired-end reads were de-multiplexed using Bc12fastq software v2.20 and FastQ files were generated based on the unique dual barcode sequences. The sequencing quality was assessed using FastQC v0.11.8 software (Andrews, 2010). Raw reads with primer sequence were processed using the Cutadapt tool (Martin, 2011). The adapter sequences were trimmed and bases above Q30 were considered, and low-quality bases were filtered off during read preprocessing and used for downstream analysis. The high-quality R1 and R2 reads were filtered and stitched using Fastq-join (Aronesty, 2013). ITS1 and ITS2 sequences were merged and considered for further analysis using the QIIME pipeline (Caporaso et al., 2010). The query sequences were clustered using the UCLUST (Edgar, 2010) method against the reference UNITE (Abarenkov et al., 2010) database (ver7) with 97% similarity. The reads, which did not hit the reference database, were then clustered using the *de novo* method where reads are clustered against one another without any external reference sequence collection. Taxonomy was assigned through the BLAST method (Altschul et al., 1990), with an *e*-value of 0.001. The BIOM file generated was taken ahead for further advanced analysis and visualization. The rarefied biom at a depth of 177,000 for merged ITS sequences/samples was used for the calculation of alpha diversity indices using various metrics. Sequences from all six agricultural management scenarios have been submitted to NCBI with the Bio project: PRJNA563827.

Biplot and principal component analysis (PCA) were done with JMP 14.1 software. The results were submitted to PCA to determine the common relationships between fungi classes, soil physical properties, and SOC, N, P, and K contents of the soil. The crop yield data were analyzed by the analysis of variance (ANOVA) technique for a completely randomized block design using SAS 9.1 software (SAS Institute, Cary, NC). Differences among treatment means were compared using Tukey's HSD test at the 5% probability level.

## Results

### Fungal diversity influenced by management scenarios

From the 18 soil samples, three of each from six scenarios, a total of 12,468,774 paired-end reads were obtained after processing of sequences, and a total of 5,033,723 sequences were obtained. Fungal diversity was calculated by different indices, such as Shannon's diversity index, Simpson's diversity index, and Chao1. Diversity indices were found to be significantly affected by agricultural management systems, as Shannon's diversity index, Simpson's diversity index, Chao1, and observed species/OTU were varied in scenarios (Table 2). Shannon diversity index was found to be 1.47 times higher in maize-based scenarios (ScIV and ScVI) as compared to rice-based CSA scenarios (ScIII and ScV). A similar pattern was observed for the Simpson index, as it was 1.12 times higher in maize-based scenarios compared with rice-based CSA scenarios. Shannon's diversity index (6.80 ± 0.551), Simpson's diversity index (0.95 ± 0.016), Chao1 (3,223 ± 256), and observed species/OTU (2,874 ± 229) were found highest in the partial CSA scenario (ScII). Our results showed ahigher Shannon diversity index (1.55 times), Simpson index (1.18 times), Chao1 (1.41 times), and observed species (1.49) in ScII than in ScIII.

**Table 2:** Diversity indices of fungi in different scenarios of crop management systems.

Scenarios*	**Shannon**	**Simpson**	**Chao1**	**Observed species/OTU**
ScI	5.71^B**^± 0.195	0.93^A^ ± 0.010	2,891^AB^ ± 96	2,529^AB^ ± 87
ScII	6.80^A^ ± 0.551	0.95^A^ ± 0.016	3,223^A^ ± 256	2,874^A^ ± 229
ScIII	4.38^C^ ± 0.150	0.85^B^ ± 0.013	2,289^C^ ± 66	1,929^C^ ± 46
ScIV	6.60^A^ ± 0.164	0.96^A^ ± 0.004	2,416^BC^ ± 132	2,087^BC^ ± 128
ScV	4.46^C^ ± 0.051	0.85^B^ ± 0.034	2,472^BC^ ± 126	2,150^BC^ ± 121
ScVI	6.42^A^ ± 0.163	0.94^A^ ± 0.028	2,077^C^ ± 220	1,829^C^ ± 222

^A*^Refer Table 1 for scenarios description.

B** Means followed by similar uppercase letters within a column are not significantly different at 0.05 level of probability using Tukey's HSD test.

**Figure 2:**
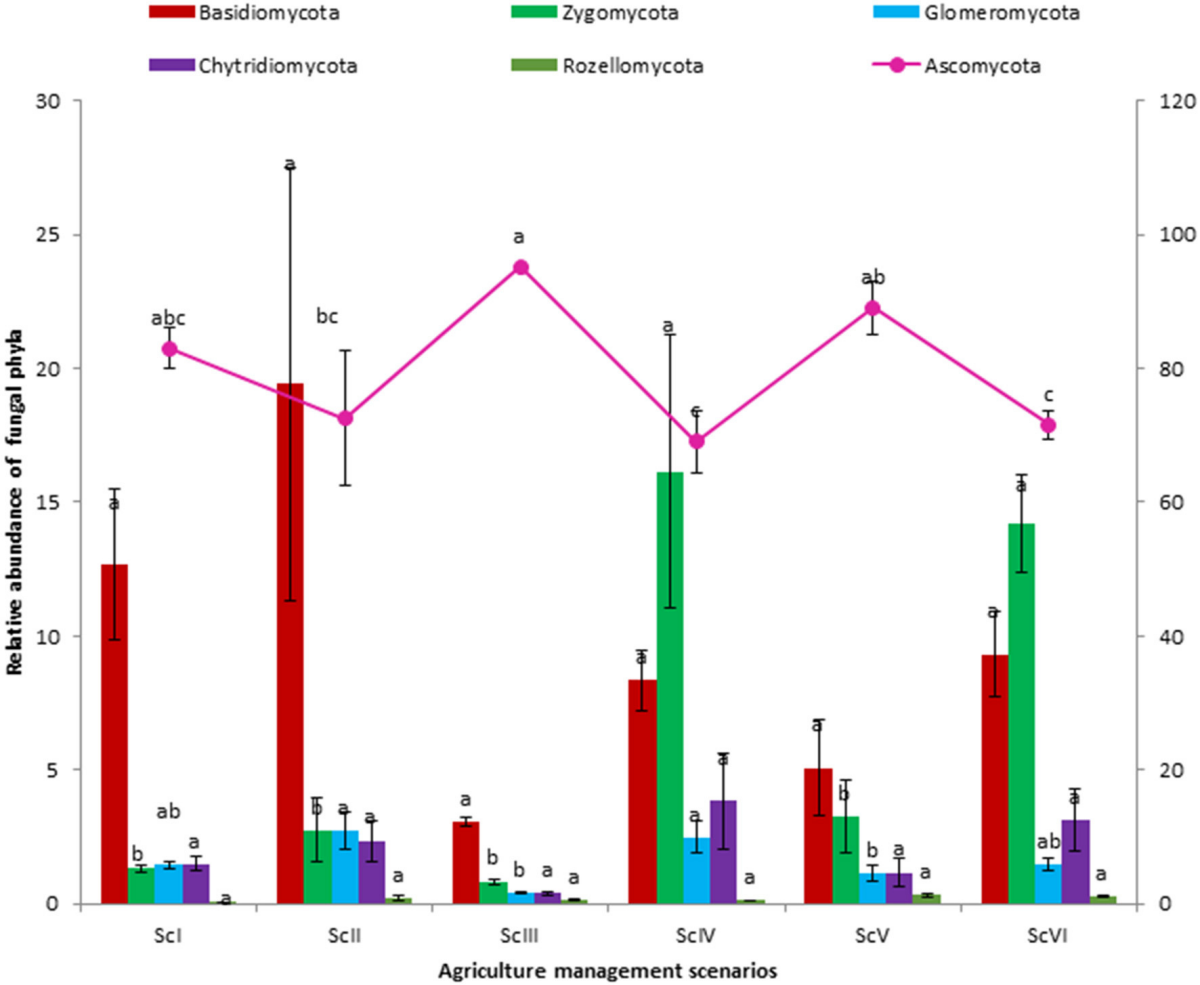
Abundance of phyla in different crop management-based scenarios. ^a,b,c^Means followed by similar lowercase letters are not significantly different at 0.05 level of probability using Tukey's HSD test.

A total of seven fungal phyla were present in all six scenarios. Ascomycota is the dominating phyla followed by Basidiomycota and Zygomycota (Figure 2). A higher abundance of Ascomycota was observed in rice-based CSA scenarios (ScIII and ScV) as compared to maize-based scenarios (ScIV and ScVI). In rice-based scenarios (ScI, ScII, ScIII, and ScV), Ascomycota was followed by Basidiomycota but in maize-based scenarios (ScIV and ScVI), it was followed by Zygomycota. Higher Ascomycota (1.31 times) was found in ScIII than ScII, whereas, Glomeromycota was found 7 times higher in ScII than ScIII and 1.66 times higher in ScIV than ScVI. Chytridiomycota was found non-significantly higher in maize-based scenarios as compared to rice-based scenarios. In all six scenarios, a total of 48 classes were observed. Sordariomycetes, an unidentified class of Ascomycota, and Dothideomycetes are among the dominating classes (Table 3). The highest abundance was observed for Sordariomycetes (25.45–43.47%), except for ScV in which an unidentified class of Ascomycota (39.30%) was found in the highest abundance. The abundance of Sordariomycetes was found at par among all the scenarios. The relative abundance of the unidentified class of Ascomycota was found to be 7.8 times higher in CSA-based rice systems compared with maize systems. Although the difference in class Agaricomycetes was non-significant among scenarios, it was two times higher in maize-based scenarios (7.72%) as compared to rice-based scenarios (3.83%). The abundance of Mortierellomycotina_cls_Incertae_sedis class of Zygomycota was recorded 8.4 times higher in maize-based scenarios than in rice-based scenarios. Ascomycota unidentified class was respectively higher in ScIII (2.72 times) than that in ScII and 1.42 times higher in ScV than ScIII. Pezizomycetes class was 6.84 times, Glomeromycetes was 6.97 times, Saccharomycetes was 3.75 times, and Blastocladiomycetes was 8.5 times higher in ScII than that in ScIII. At the level of order, Hypocreales was the dominating order followed by Pleosporales, Pezizales, and Sordariales (Supplementary Table 1).

**Table 3:** Relative abundance of classes in different crop management scenarios.

Scenarios*	**Sordariomycetes**	**Ascomycota;unidentified **	**Dothideomycetes**	**Agaricomycetes**	**Eurotiomycetes**	**Pezizomycetes**	**Tremellomycetes**	**Glomeromycetes**	**Saccharomycetes**	**Mucoromycotina_cls** **_Incertae_sedis **	**Chytridiomycetes**	**Mortierellomycotina_cls** **_Incertae_sedis **	**Chytridiomycota;** **unidentified **	**Blastocladiomycetes **	**Pezizomycotina_cls** **_Incertae_sedis **
ScI	29.55	21.65^B**^	22.84	10.48	3.17^B^	4.00^AB^	1.66	1.46^AB^	1.39^A^	0.61^A^	0.97	0.65^B^	0.31	0.19^A^	0.15
ScII	34.54	10.21^C^	13.47	8.79	6.67^A^	5.06^A^	10.12	2.72^A^	1.05^AB^	0.16^B^	1.81	2.53^B^	0.32	0.17^AB^	0.4
ScIII	42.65	27.77^B^	21.43	2.42	1.86^B^	0.74^C^	0.47	0.39^B^	0.28^C^	0.08^B^	0.2	0.72^B^	0.16	0.02^C^	0.09
ScIV	31.37	3.85^C^	22.19	7.14	3.71^B^	5.07^A^	0.65	2.47^A^	1.08^AB^	0.66^A^	2.27	15.45^A^	1.51	0.05^C^	1.3
ScV	25.45	39.30^A^	19.91	4.23	1.06^B^	1.94^BC^	0.55	1.12^B^	0.37^C^	0.19^B^	0.72	3.05^B^	0.33	0.09^BC^	0.13
ScVI	43.47	4.72^C^	14.45	8.03	2.90^B^	3.95^AB^	0.78	1.48^AB^	0.74^BC^	0.42^AB^	2.57	13.74^A^	0.47	0.08^BC^	0.8
Mean	34.5	17.92	19.05	6.85	3.23	3.46	2.37	1.61	0.82	0.35	1.42	6.02	0.52	0.1	0.48
p-value	0.3821	< 0.0001	0.4627	0.0599	0.0189	0.0413	0.3542	0.0267	0.0198	0.0176	0.2938	0.0013	0.1215	0.0242	0.0866
CV(%)	33.47	26.66	36.92	43	48.27	46.45	249.18	45.8	43.28	55.69	94.99	61.98	108.81	56.13	107.04
SE(d)	9.428	3.9	5.742	2.404	1.273	1.312	4.822	0.601	0.29	0.16	1.105	3.048	0.46	0.046	0.416
LSD at 5%	NS	8.6907	NS	NS	2.836	2.9242	NS	1.34	0.6453	0.3567	NS	6.792	NS	0.1014	NS

* Refer Table 1 for scenarios description.

** Means followed by similar uppercase letters within a column are not significantly different at 0.05 level of probability using Tukey's HSD test.

### Effect of agriculture management scenarios on soil chemical and physical properties

Differences among the scenarios were recorded for soil chemical and physical parameters. Soil organic carbon (SOC) and available nitrogen (N) were 1.43 and 1.34 times higher in CSA-based scenarios (ScII, ScIII, ScIV, ScV, and ScVI) compared with farmers' practice (ScI). Available phosphorus and potassium were found to be 1.65 and 1.60 times higher, respectively, in the soil of CSA-based scenarios than that in the CT scenario/farmers' practice. All four SOC (5.7 ± 0.01 g kg^−1^), N (119.01 ± 0.58 kg ha^−1^), P (16.1 ± kg ha^−1^), and K (137 ± kg ha^−1^) were lowest with farmers' practice (ScI). Bulk density was observed highest in ScI (1.58) followed by ScII (1.52) (Table 4). It was lower in rice-based CSA scenarios (1.36) as compared to maize-based scenarios (1.445). The highest mean weight diameter was found in ScIII (2.95 mm) and ScV (2.70 mm) and the lowest was observed in ScI (1.45 mm). Water-stable aggregate was non-significant in full/CSA scenarios but significantly higher in ScII (68.3%) and farmers' practice (62.7%) than that in other scenarios.

**Table 4:** Effect of management practices on soil chemical and physical properties and grain yield.

Scenarios*	**SOC** **(g kg^−1^)**	**N** **(kg ha^−1^)**	**P** **(kg ha^−1^)**	**K** **(kg ha^−1^)**	**BD**	**WSA** **(%)**	**MWD** **(mm)**	**Grain yield (Mg ha** ^**−1**^ **)**		
								**Rice/maize**	**Wheat**	**System**
ScI	5.7^D**^	119^D^	16.1^C^	137^C^	1.58^A^	62.7^C^	1.45^D^	6.37^BC^	5.88^C^	13.33^C^
ScII	7.1^C^	144^C^	23.1^B^	226^A^	1.52^B^	68.3^B^	1.81^C^	6.63^B^	6.06^BC^	16.19^AB^
ScIII	9.0^A^	171^A^	24.9^B^	218^B^	1.35^D^	75.7^A^	2.95^A^	6.60^B^	6.58^AB^	14.86^BC^
ScIV	8.3^AB^	156^B^	29.7^A^	226^A^	1.43^C^	72.5^A^	2.50^B^	6.85^B**^	6.49^AB^	16.04^AB^
ScV	8.1^B^	171^A^	30.5^A^	210^B^	1.37^D^	76.3^A^	2.70^A^	6.86^B^	6.61^AB^	15.61^AB^
ScVI	8.5^AB^	157^B^	24.2^B^	218^B^	1.46^C^	73.7^A^	2.28^B^	7.67^B**^	6.79^A^	16.85^A^

WSA, water stable aggregates; MWD, mean weight diameter.

* Refer Table 1 for scenarios description.

** Means followed by similar uppercase l.etters within a column are not significantly different at 0.05 level of probability using Tukey's HSD test.

### Crop and system yield

The total residue load in ScI, ScII, ScIII, ScIV, ScV, and ScVI was 105, 101, 119, 101, and 119 Mg ha^−1^, respectively. During the year 2018, a higher yield of wheat was recorded with all the CSA-based scenarios (ScIII–ScVI) and ranged from 6.58 to 6.79 Mg ha^−1^. The lowest yield was recorded with farmers' practice, that is, 5.88 Mg ha^−1^. A similar trend was also observed for the system yield in all the scenarios (Table 4). The higher system yield was recorded with ScVI (16.85 Mg ha^−1^) followed by ScII (16.19 Mg ha^−1^) and ScIV (16.04 Mg ha^−1^). The lowest yield was found with farmers' practice or ScI (13.33 Mg ha^−1^).

### Correlations between soil properties

**Figure 3:**
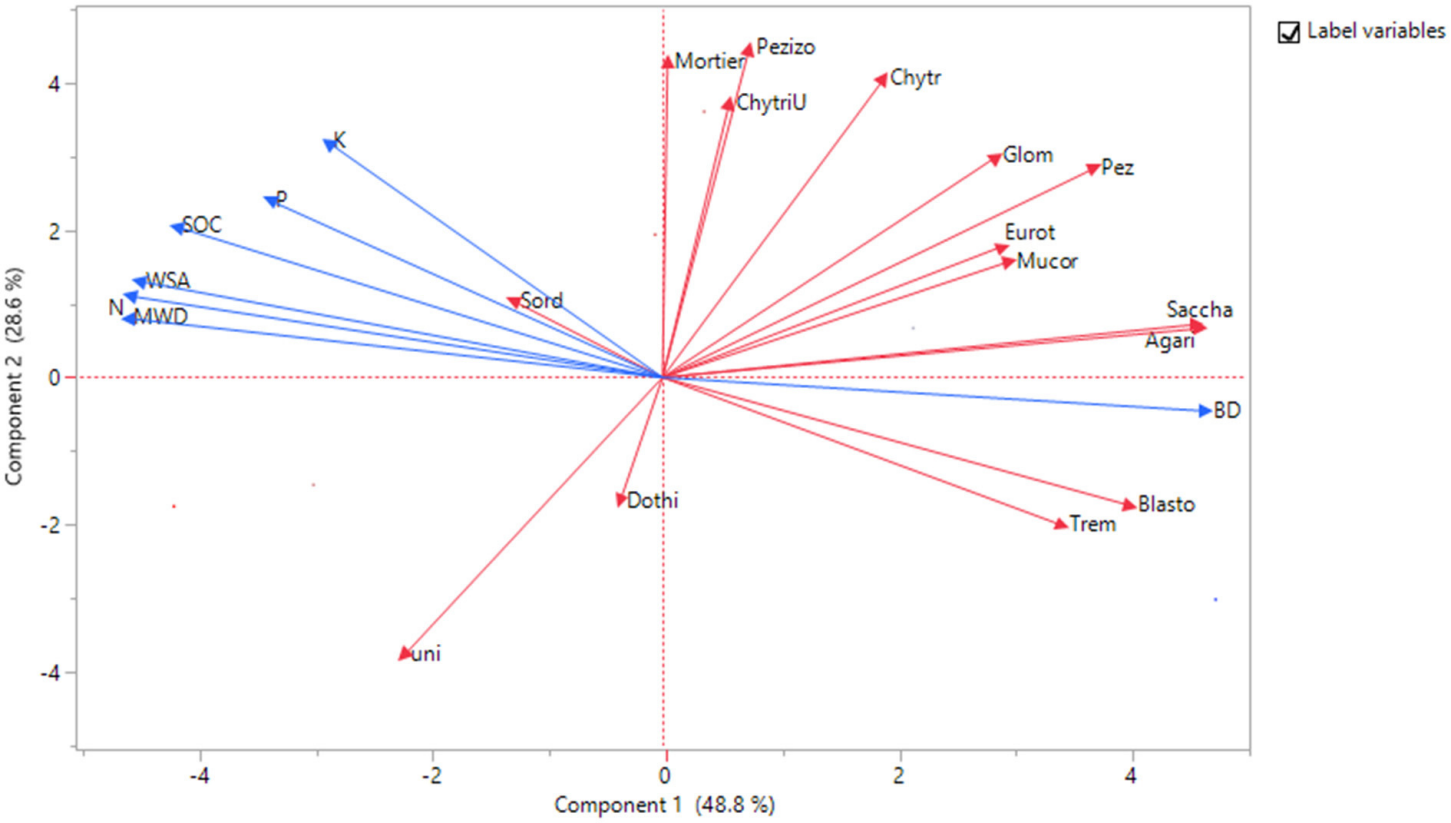
Biplot obtained from principal components analysis based on the correlation matrix, showing the two first principal components (explaining 48.8 and 28.6%, respectively). Each point represents scenarios (ScI to ScVI), loadings represent fungi classes, soil physical properties, and SOC, *N, P*, and *K* contents of the soil.

The results from the principal component analysis provided evidence that the different scenarios were diverse regarding the fungi classes, soil physical properties, and SOC, N, P, and K contents of the soil (Figure 3). According to the factor loadings, the first PC, which explains 48.8% of the total variance, had higher positive correlations with Agaricomycetes, Saccharomycetes, Blastocladiomycetes, and bulk density (BD), and a negative correlation with SOC, N, WSA, and MWD, while the second PC, which explains 16.76% of the total variance, was strongly correlated with Chytridiomycetes, Mortierellomycotina_cls_Incertae_sedis, and Pezizomycotina_cls_Incertae_sedis. The third PC explains 12.6% of the total variance and is correlated with Dothideomycetes (Supplementary Table 2). The PCA biplot in Figure 3 shows both PC scores of samples and the loadings of variables.

Significant correlations were observed among the fungi classes, soil physical properties, and SOC, N, P, and K contents of soil irrespective of scenarios (Table 5). An unidentified class of fungi was significantly negatively correlated to Pezizomycetes, Chytridiomycetes, and Pezizomycotina_cls_Incertae_sedis. Dothideomycetes was significantly negatively correlated to WSA and MWD. Agaricomycetes was significantly positively correlated to Pezizomycetes, Saccharomycetes, and Blastocladiomycetes, and negatively correlated to the available N content of the soil. Eurotiomycetes was significantly positively correlated to Pezizomycetes, Tremellomycetes, and Glomeromycetes. Pezizomycetes was significantly positively correlated with Glomeromycetes, Saccharomycetes, and Chytridiomycetes. Glomeromycetes was positively significantly correlated to Chytridiomycetes. Saccharomycetes was positively correlated to Mucoromycotina_cls_Incertae_sedis and Blastocladiomycetes, and negatively correlated to N. Mucoromycotina_cls_Incertae_sedis, which was positively correlated to Chytridiomycota;unidentified. Chytridiomycetes was positively correlated to Mortierellomycotina_cls_Incertae_sedis, Pezizomycotina_cls_Incertae_sedis, and WSA. Mortierellomycotina_cls_Incertae_sedis was significantly positively correlated to Chytridiomycota;unidentified and Pezizomycotina_cls_Incertae_sedis, and WSA. Chytridiomycota;unidentified was significantly positively correlated to Pezizomycotina_cls_Incertae_sedis. Blastocladiomycetes was positively correlated to BD and negatively correlated to the C, N, and P contents of the soil. WSA was significantly positively correlated to MWD and C and K contents. MWD was positively correlated to the C and K contents of the soil. BD was significantly negatively correlated to C, N, P, and K contents of the soil. Soil carbon was positively correlated to the N, P, and K contents of the soil. The available N was significantly positively correlated with available P and K content of the soil. The available P was significantly positively correlated to the available K content of the soil.

**Table 5:** Correlation between fungal class abundance, soil chemical and physical properties in different management scenarios.

	**Sord**	**Unid**	**Dothi**	**Agari**	**Eurot**	**Pez**	**Trem**	**Glom**	**Saccha**	**Mucor**	**Chytr**	**Mortier**	**ChytriU**	**Blasto**	**Pezizo**	**WSA**	**MWD**	**BD**	**C**	**N**	**P**	**K**
Sord	1																					
Unid	−0.40																					
Dothi	−0.39	0.40																				
Agari	−0.19	−0.57	−0.23																			
Eurot	0.06	−0.65	−0.54	0.61																		
Pez	−0.18	–0.79*	−0.33	0.84*	0.77*																	
Trem	−0.03	−0.27	−0.64	0.40	0.89*	0.49																
Glom	−0.27	−0.68	−0.38	0.64	0.83*	0.93**1	0.64															
Saccha	−0.29	−0.56	0.04	0.93**1	0.62	0.84*	0.34	0.68														
Mucor	−0.27	−0.51	0.38	0.64	0.07	0.62	−0.31	0.39	0.74*													
Chytr	0.18	−0.89*	−0.52	0.56	0.50	0.81*	0.20	0.72*	0.46	0.51												
Mortier	0.20	−0.72	−0.16	0.16	0.06	0.49	−0.28	0.41	0.14	0.56	0.84*											
ChytriU	−0.20	−0.57	0.26	0.15	0.15	0.55	−0.21	0.55	0.34	0.67*	0.59	0.79*										
Blasto	−0.42	−0.05	−0.23	0.80*	0.54	0.51	0.6	0.43	0.71*	0.19	0.08	−0.4	−0.30									
Pezizo	0.11	−0.81*	−0.09	0.26	0.28	0.65	−0.10	0.60	0.33	0.61	0.84*	0.94**	0.90*	−0.29								
WSA	0.4	−0.52	–0.71*	−0.07	0.18	0.28	0.11	0.33	−0.25	−0.10	0.72*	0.72*	0.31	−0.36	0.60							
MWD	0.19	−0.33	–0.70*	−0.11	0.1	0.21	0.12	0.30	−0.32	−0.18	0.62	0.61	0.22	−0.28	0.47	0.96**						
BD	−0.18	−0.42	−0.18	0.96**	0.62	0.71	0.48	0.53	0.91*	0.51	0.34	−0.09	−0.05	0.89*	0.02	−0.28	−0.32					
C	0.51	−0.06	−0.11	−0.78	−0.39	−0.46	−0.38	−0.33	−0.79	−0.39	0.04	0.41	0.18	–0.94**	0.28	0.61*	0.55*	–0.89*				
N	0.25	0.29	−0.07	−0.89*	−0.49	−0.59	−0.33	−0.39	−0.91*	−0.56	0.17	0.18	0.03	–0.84*	0.05	0.49	0.53	–0.75*	0.88*			
P	−0.16	0.11	0.01	−0.63	−0.3	−0.18	−0.26	0.04	−0.57	−0.20	0.10	0.44	0.47	–0.69*	0.39	0.54	0.62	–0.81*	0.94**	0.83*		
K	0.36	−0.26	−0.48	−0.49	0.16	−0.02	0.18	0.21	−0.52	−0.44	0.32	0.43	0.29	−0.61	0.42	0.79*	0.75*	–0.88*	0.79*	0.76*	0.76*	1

Sor, Sordariomycetes, Unid, Ascomycota; unidentified, Dothi, Dothideomycetes, Agari, Agaricomycetes, Eurot , Eurotiomycetes, Pez, Pezizomycetes, Trem, Tremellomycetes, Glom, Glomeromycetes, Saccha, Saccharomycetes, Mucor, Mucoromycotina_cls_Incertae_sedis, Chytr, Chytridiomycetes, Mortier, Mortierellomycotina_cls_Incertae_sedis, ChytriU, Chytridiomycota; unidentified, Blasto, Blastocladiomycetes, Pezizo, Pezizomycotina_cls_Incertae_sedis, WSA, water-stable aggregates, MWD, Mean weight diameter, BD, bulk density, C, organic carbon, P, available phosphorus, N, available nitrogen, K, available potassium.

* Correlation is significant at the 0.05 level;

** Correlation is significant at the 0.01 level;

^***^Correlation is significant at the 0.001 level.

## Discussion

Conservation agriculture-based practices showed higher Shannon and Simpson diversity indices in maize-based scenarios as compared to rice-based scenarios due to the inclusion of maize in the crop rotation system. Although sampling of soil was done in all scenarios after harvesting the common crop among them (wheat), the residue and root system of the previous crop can impact soil properties. Fungal diversity is directly or indirectly affected by plant and soil properties (Yang et al., 2017). Maize has a different root system than rice (tap root instead of fibrous root system); the amount and composition of root exudates vary with crops/plants, which can influence the microbial diversity of soil (Sasse et al., 2018). Moreover, the quantity and quality of residue also play a critical role in the microbial properties of soil (Moore et al., 2000). Maize residue provides higher amounts of lignocellulosic material, which can harbor more types of residue-decomposing fungi, hence showing higher fungal diversity than rice-based CA practices. Similar results were reported in our previous study (Choudhary et al., 2018b) based on 5 years of continuous CA-based practices. This combination of residue retention and incorporation in one calendar year may be the reason for the highest diversity indices in this scenario II instead of only crop residue retention in a similar cropping system. Through the tillage practices, crop residues mix well in the soil and alter the microclimate and distribution of nutrients, which resulted in higher fungal diversity under partial CA practice than that in other practices where only crop residues were retained.

Ascomycota, found as a dominating phylum in all management scenarios, is well known as one of the most abundant fungal phyla of soils globally (Choudhary et al., 2018a,b; Egidi et al., 2019; Maguire et al., 2020). Ascomycota is the largest phylum of fungi and is ubiquitous in soil (Money, 2016; Egidi et al., 2019). Most Ascomycota members are saprophytic and the main decomposers of plant residue in the soil, hence they dominate the fungal community composition in soils. In our study, a higher abundance of Ascomycota was observed in rice-based CA scenarios as compared to maize-based scenarios due to the difference between residues. Since soil samples were taken after the harvesting of the wheat crop (common crop in all scenarios) and by that time ample duration (5 months for maize and rice residue) has been received by the residue to decompose. Initially, the residue decomposes fast due to the presence of water-soluble compounds in the residues, which are easily decomposable (Diochon et al., 2016), but in later stages, decomposition of more recalcitrant compounds such as lignin and cellulose takes place. The composition of residue and decomposition, which varies with crop type, are the main factors in harboring different types of microbial communities. This might also lead to the high abundance of Basidiomycota in rice-based scenarios and Zygomycota in maize-based scenarios. The type of crop, size, and type of residue and tillage practices vary in different management systems leading to variation in soil moisture also. Chytridiomycota are reported in aquatic ecosystems as well as in terrestrial ecosystems (Gleason et al., 2004), but in our study, these were found in different management systems and favored in maize-based scenarios. Not only residue type and decomposition duration but also variation in moisture played an important role in deciding the abundance of different groups of fungi (Miura et al., 2015). Sordariomycetes and Eurotiomycetes were among the dominating classes in all the scenarios, which were previously reported in soils of conservation agricultural practices (Wang et al., 2016; Choudhary et al., 2018b). Sordariomycetes is one of the largest classes of Ascomycota, which includes pathogens, endophytes, and saprobes. Members of the Sordariomycetes are ubiquitous and cosmopolitan. Saprobic Sordariomycetes have the potential to produce cellulolytic enzymes, and its member *Chaetomium* is a well-known cellulolytic organism responsible for the destruction of paper and fabrics (Zhang et al., 2006). Members of Sordariomycetes class play an important role in the decomposition and nutrient cycling of plant residues. Dothideomycetes also represents one of the largest and dominant classes of the phylum Ascomycota, which is mostly found as endophytes or saprobes (Kirk et al., 2008). Members of this class can cause disease in almost every crop plant including maize and rice (Goodwin, 2013). Members of this class are also known to degrade cellulose and other complex carbohydrates of dead or partially digested plant organic matter (Hyde et al., 2013). One unidentified class of Ascomycota is also among the most dominating classes found in scenarios. Although its identification is not known to class level, these unidentified and unknown fungal groups may be important to community compositions. Many of these previously unknown groups are later found as major components of communities of interest (Rosling et al., 2011). The abundance of some classes found to be high in maize-based scenarios can be correlated with the difference in crop type, root system, and residue depending on plant species, and biochemical characteristics of plant materials differ considerably (Xu et al., 2006). The effect of tillage practices on the abundance of fungi was observed at both phyla and class levels. Some phyla and classes were found higher in ZT, which indicates that the hyphal network in CT practices got disturbed due to intensive tillage practices, which resulted in limited growth. Whereas, the abundance of some phyla/classes was reported higher in CT than ZT, which might be due to the higher growth rate of broken mycelia of these phyla/classes under CT than that in other management practices. Wang et al. (2017) concluded that tillage influenced the distribution of fungal communities with more stable communities under ZT conditions. Irrigation management and soil moisture had a great impact on fungal communities in arid and semi-arid areas where water availability is limited (Vargas-Gastélum et al., 2015; Deng et al., 2022). But in our study, irrigation was given to all the scenarios based on the tensiometer to maintain the soil moisture as per crop requirement to minimize the effect of irrigation management on fungal community composition.

Crop residue retention enriches soils with a significant amount of nutrients (Jat et al., 2018, 2019b; Lohan et al., 2018). It is a major reason behind high SOC, N, P, and K contents in residue retention scenarios. Moreover, under zero tillage practices, the decomposition of residue also does not disturb due to the non-disturbance of soil. Residue not only harbors different types of microfauna but also provides nutrients to them, which in turn release different types of enzymes such as phosphatase, glucosidase, dehydrogenase, etc., responsible for the conversion of unavailable to the available forms of nutrients (Maguire et al., 2020). The activity of different enzymes is reported to be more in CA/CSA-based practices and have a higher soil quality index over farmers' practices (Choudhary et al., 2018c,d). CA practices not only improve nutrient availability but also improve overall soil quality (Jat et al., 2021a). It was observed by many researchers that tillage and residue had a positive effect on bulk density (Gathala et al., 2011). In farmers' practice, the residue is either burnt or removed, which resulted in more compactness in soils. Lower BD in the rice-based system as compared to maize-based CA scenarios might be due to higher conversion of crop residue carbon to SOC leading to good soil structure as evidenced through higher WSA and MWD (Mandal et al., 2007).

The results clearly showed the differential benefits of CSA-based management practices in both rice- and maize-based systems. The highest wheat and systems yield (rice equivalent yield basis) were recorded with all CSA-based scenarios. Tillage and cropping systems, crop residue management, soil type, and climate control the magnitude at which SOC affects crop yields (Blanco-Canqui et al., 2013). About 0.1% increase in SOC concentration enhanced wheat yield by 0.04 Mg ha^−1^ at 0 kg N ha^−1^ (Blanco-Canqui et al., 2012). A direct relationship between SOM stocks and crop productivity was observed by Oldfield et al. (2018) through variables for soils and amendments. In a global meta-analysis, zero tillage (ZT) when combined with residue retention and crop rotation can produce equivalent or greater yields than conventional tillage, by minimizing its negative impacts. Higher system productivity in CSA-based scenarios might be due to the integration of mungbean (Gathala et al., 2013), less terminal heat effects on the wheat crop (Gathala et al., 2013; Sharma et al., 2015), higher carbon mineralization (Kirk et al., 2008; Datta et al., 2019) with better nutrient availability (Sasse et al., 2018), and improved soil biological properties (Olsen et al., 1954; Choudhary et al., 2018b,d).

Some classes are negatively correlated while some are positively correlated with each other, and it is due to the ecological relationship between them, which may influence each other's position in the community. The groups, which have similar modes of nutrition, compete for the same biochemical fraction of the crop residues, and this might show a negative correlation. Biochemical qualities of residue are the main drivers in the composition of fungal communities. Specific function during the decomposition of residue linked to specific taxa (Rezgui et al., 2021). In residue decomposition, the end product of one class/group may be acted as food for another group. In such a situation, these may be positively correlated as they do not have competition for food. Due to the preferences of microbes for specific residue compounds, organic matter decomposition and transformation under different agricultural managements influence the abundances of the specific microbial taxa (Zheng et al., 2022). Different type of residue management over the years strongly influences the soil's microbial, chemical, and physical properties. In this 9-year-old experiment, the residue is accumulated over the years in all CSA scenarios, and according to Fontaine et al. (2011), the soil microbes can decompose old recalcitrant soil organic matter by using fresh carbon as a source of energy, which can lead to a different type of distribution of soil fungal taxa in these scenarios. A significant positive correlation between SOC and MWD and WSA was due to good soil structure rendered by higher SOC in CA-based scenarios. Higher MWD and WSA were found under crop residue retention scenarios in cereal systems (Jat et al., 2019a). There is a strong link between microbial communities and SOC (Zheng et al., 2022), which is influenced by the different tillage and crop establishment practices. ZT improved C fractions over CT and these C pools can directly impact the activities of microbes in soils (Rakesh et al., 2021). Soil properties and soil fungal communities also showed different types and magnitudes of relationships because of robust associations between them (Yang et al., 2017). Both the diversity and composition of the fungi community directly correlated with the soil properties and indirectly correlated with management practices (Li and Zhang, 2022).

## Conclusion

Agricultural practices in isolation do not prove good for soil and crop sustainability in cereal systems. Crop management practices, such as tillage, residue management, nutrient, water management, and crop diversification have a strong influence on the physical, chemical, and biological properties of soil. Therefore, the combination of different management practices led to differences in fungal community composition and soil chemical properties under climate-smart agriculture (CSA) systems. Maize-based scenarios are more diverse in terms of fungal communities than rice-based CSA scenarios. Rice-based scenarios with CSA practices showed a more abundance of Ascomycota phyla over the maize-based CSA scenarios. Ascomycota is followed by Basidiomycota in rice-based scenarios and Zygomycota in maize-based scenarios. CSA-based scenarios improved the soil chemical properties (organic carbon, nitrogen, and potassium) across the different cereal-based CSA scenarios compared with other scenarios (CT and partial CSA). Soil physical properties such as bulk density, mean weight diameter, and water-stable aggregate were also improved under CSA scenarios. Improved soil properties under CSA-based practices resulted in improved crop and system yield. Results indicate that the bundling/layering of smart agricultural practices not only influences the soil properties but also played an important role in deciding the microbial community composition.

## Data availability statement

The datasets presented in this study can be found in online repositories. The names of the repository/repositories and accession number(s) can be found at: https://www.ncbi.nlm.nih.gov/, PRJNA563827.

## Ethics statement

The experiment was conducted after taking proper approval from the Institute Research Committee of ICAR-CSSRI, Karnal. Guidelines of the ICAR-CSSRI were followed for taking data on crops/weeds/plants.

## Author contributions

HJ, PS, and MJ conceptualized and designed the experiment. MJ and PS did the funding acquisition. HJ and PS did the management of the experiment. HJ and MC conducted the research. MC wrote the manuscript. HJ provided critical comments on the manuscript. All authors read and approved the final manuscript.
