# Supplementary material for: Climate-smart agricultural practices influence the fungal communities and soil properties under major agri-food systems
Source: Front Microbiol. 2022 Dec 13;13:986519. doi: 10.3389/fmicb.2022.986519 (PMC9794093; doi:10.3389/fmicb.2022.986519)
Supplement: Supplementary file 1 [file Data_Sheet_1.docx]

| **Scenarios** | Hypocreales | Pleosporales | Pezizales | Sordariales | Eurotiales | Agaricales | Tremellales | Sordariomycetes; unidentified | Russulales | Polyporales | Cantharellales | Saccharomycetales | Myriangiales |
| --- | --- | --- | --- | --- | --- | --- | --- | --- | --- | --- | --- | --- | --- |
| **ScI** | 28.9 | 26.66 | 5.11 | 3.64^BC^ | 3.21^A^ | 2.87 | 2.05 | 4.72^B^ | 2.12 | 3.22 | 1.34 | 1.77^A^ | 1.07^B^ |
| **ScII** | 30.54 | 11.17 | 5.66 | 2.74^BC^ | 1.76^AB^ | 3.4 | 10.95 | 4.68^B^ | 1.08 | 0.14 | 1.09 | 1.19^AB^ | 3.17^A^ |
| **ScIII** | 52.92 | 27.78 | 0.99 | 1.34^C^ | 0.34^B^ | 1.19 | 0.6 | 4.18^B^ | 0.66 | 0.02 | 0.13 | 0.39^C^ | 0.52^B^ |
| **ScIV** | 24.3 | 17.34 | 5.31 | 4.92^B^ | 1.83^AB^ | 1.35 | 0.66 | 2.65^B^ | 0.85 | 0.07 | 0.22 | 1.15^AB^ | 0.97^B^ |
| **ScV** | 19.63 | 30.95 | 3.14 | 8.44^A^ | 1.30^B^ | 1.71 | 0.82 | 12.77^A^ | 0.96 | 0.04 | 0.67 | 0.60^BC^ | 0.98^B^ |
| **ScVI** | 36.39 | 11.25 | 3.35 | 5.90^AB^ | 1.33^B^ | 1.99 | 0.8 | 2.21^B^ | 1.34 | 0.17 | 0.2 | 0.79^BC^ | 1.00^B^ |
| **General Mean** | 32.11 | 20.86 | 3.92 | 4.5 | 1.63 | 2.09 | 2.65 | 5.2 | 1.17 | 0.61 | 0.61 | 0.98 | 1.29 |
| **p-Value** | 0.1181 | 0.1288 | 0.0855 | 0.0094 | 0.0417 | 0.1454 | 0.3535 | 0.0124 | 0.1729 | 0.1406 | 0.1588 | 0.018 | 0.0077 |
| **CV(%)** | 41.13 | 48.38 | 47.92 | 40.32 | 53.13 | 49.81 | 239.44 | 55.78 | 54.92 | 246.46 | 103.37 | 40.26 | 51.63 |
| **SE(d)** | 10.784 | 8.24 | 1.535 | 1.481 | 0.706 | 0.848 | 5.175 | 2.37 | 0.524 | 1.231 | 0.512 | 0.322 | 0.542 |
| **LSD at 5%** | NS | NS | NS | 3.3003 | 1.5721 | NS | NS | 5.2812 | NS | NS | NS | 0.7184 | 1.2078 |

Supplementary table S1. Relative abundance of different fungal orders in agriculture management scenarios.

| **Chaetothyriales** | **Capnodiales** | **Boletales** | **Mortierellales** | **Diversisporales** | **Mucorales** | **Glomerales** | **Rhizophydiales** | **Auriculariales** | **Sebacinales** | **Helotiales** | **Dothideomycetes; unidentified** | **Pezizomycotina_ord_Incertae_sedis** |
| --- | --- | --- | --- | --- | --- | --- | --- | --- | --- | --- | --- | --- |
| 0.66^B^ | 1.51 | 1.13^BC^ | 0.83^B^ | 0.99^A^ | 0.78^A^ | 0.67^B^ | 0.48 | 0.34^B^ | 0.29^B^ | 0.06 | 0.09^C^ | 0.19 |
| 5.21^A^ | 0.56 | 2.21^B^ | 2.79^B^ | 0.81^AB^ | 0.30^BC^ | 1.94^A^ | 1.2 | 0.98^B^ | 0.77^A^ | 0.92 | 0.11^C^ | 0.45 |
| 2.24^B^ | 1.37 | 0.64^C^ | 1.01^B^ | 0.17^B^ | 0.11^C^ | 0.21^B^ | 0.13 | 0.56^B^ | 0.10^B^ | 0.38 | 0.08^C^ | 0.12 |
| 1.57^B^ | 0.58 | 1.68^BC^ | 16.37^A^ | 1.24^A^ | 0.69^AB^ | 0.75^B^ | 0.5 | 3.10^A^ | 0.23^B^ | 0.21 | 3.38^A^ | 1.36 |
| 0.39^B^ | 2.19 | 1.87^B^ | 4.85^B^ | 0.71^AB^ | 0.30^BC^ | 0.62^B^ | 0.44 | 1.39^B^ | 0.10^B^ | 0.02 | 0.20^C^ | 0.21 |
| 1.69^B^ | 0.71 | 3.64^A^ | 14.60^A^ | 0.25^B^ | 0.43^ABC^ | 1.09^AB^ | 0.41 | 1.64^B^ | 0.22^B^ | 0.07 | 2.12^B^ | 0.84 |
| 1.96 | 1.15 | 1.86 | 6.74 | 0.69 | 0.44 | 0.88 | 0.53 | 1.34 | 0.29 | 0.28 | 1 | 0.53 |
| 0.0242 | 0.2164 | 0.0044 | 0.0023 | 0.0289 | 0.0383 | 0.0224 | 0.1267 | 0.0145 | 0.0133 | 0.4141 | <.0001 | 0.102 |
| 74.07 | 74.43 | 36.05 | 61.47 | 51.93 | 52.4 | 55.49 | 77.73 | 57.45 | 66.13 | 200.29 | 32.76 | 100.68 |
| 1.185 | 0.701 | 0.548 | 3.384 | 0.295 | 0.187 | 0.398 | 0.335 | 0.627 | 0.154 | 0.456 | 0.266 | 0.434 |
| 2.6408 | NS | 1.2205 | 7.539 | 0.6564 | 0.4177 | 0.8875 | NS | 1.3964 | 0.343 | NS | 0.5932 | NS |

Supplementary table S2. Summarization of Principal Component Analysis.

|  |  | **PC1** | **PC2** | **PC3** |
| --- | --- | --- | --- | --- |
| Eigenvalue |  | 10.74 | 6.30 | 2.77 |
| Variability (%) |  | 48.84 | 28.34 | 12.60 |
| Cumulative % |  | 48.84 | 77.49 | 90.10 |
| Sord | Factor loadings | -0.27014 | 0.21925 | -0.41926 |
| uni |  | -0.46731 | -0.79555 | 0.13518 |
| Dothi |  | -0.07701 | -0.35542 | 0.85948 |
| Agari |  | 0.97305 | 0.14038 | -0.05828 |
| Eurot |  | 0.61486 | 0.37584 | -0.57479 |
| Pez |  | 0.78113 | 0.59918 | -0.07037 |
| Trem |  | 0.72414 | -0.42446 | 0.44298 |
| Glom |  | 0.60493 | 0.63351 | -0.19798 |
| Saccha |  | 0.96811 | 0.15240 | 0.14040 |
| Mucor |  | 0.62934 | 0.33334 | 0.68218 |
| Chytr |  | 0.40135 | 0.85769 | -0.09846 |
| Mortier |  | 0.00886 | 0.90505 | 0.29421 |
| ChytriU |  | 0.12055 | 0.78454 | 0.55118 |
| Blasto |  | 0.84639 | -0.36683 | -0.27638 |
| Pezizo |  | 0.15675 | 0.94229 | 0.28559 |
| SOC |  | -0.87907 | 0.43162 | -0.02305 |
| N |  | -0.96456 | 0.23364 | -0.04821 |
| P |  | -0.71031 | 0.50703 | 0.16950 |
| K |  | -0.60577 | 0.67469 | -0.38041 |
| BD |  | 0.98308 | -0.09617 | -0.13675 |
| WSA |  | -0.94617 | 0.27550 | 0.00593 |
| MWD |  | -0.96815 | 0.16940 | 0.16754 |
